# The Effects of a Competitor on the Foraging Behaviour of the Shore Crab *Carcinus maenas*

**DOI:** 10.1371/journal.pone.0093546

**Published:** 2014-04-01

**Authors:** Leela J. Chakravarti, Peter A. Cotton

**Affiliations:** Marine Biology and Ecology Research Centre, University of Plymouth, Plymouth, Devon, United Kingdom; University of Tours, France

## Abstract

Optimal Diet Theory suggests that individuals make foraging decisions that maximise net energy intake. Many studies provide qualitative support for this, but factors such as digestive constraints, learning, predation-risk and competition can influence foraging behaviour and lead to departures from quantitative predictions. We examined the effects of intraspecific competition within a classic model of optimal diet – the common shore crab, *Carcinus maenas,* feeding on the mussel, *Mytilus edulis*. Unexpectedly, we found that breaking time (*Tb*), eating time (*Te*), and handling time (*Th*) all decreased significantly in the presence of a conspecific. Reduced handling time in the presence of a competitor resulted in an increased rate of energy intake, raising the question of why crabs do not always feed in such a way. We suggest that the costs of decreased shell breaking time may be increased risk of claw damage and that crabs may be trading-off the potential loss of food to a competitor with the potential to damage their claw whilst breaking the shell more rapidly. It is well documented that prey-size selection by crabs is influenced by both the risk of claw damage and competition. However, our results are the first to demonstrate similar effects on prey handling times. We suggest that crabs maximise their long-term rate of energy intake at a scale far greater than individual foraging events and that in order to minimise claw damage, they typically break shells at a rate below their maximum. In the presence of a competitor, crabs appear to become more risk-prone and handle their food more rapidly, minimising the risk of kleptoparasitism.

## Introduction

Prey choice has a significant impact on individuals and may have consequences for both predator and prey populations. Since the pioneering work of Emlen [1] and MacArthur and Pianka [2], optimality theory has been used to explain prey selection as decisions involving the trade-off between costs (*e.g*., handling time) and benefits (*e.g*., energy in prey) in order to maximise the rate of net energy intake [3]. Optimal Diet Theory [4], [5] predicts that foraging predators that aim to maximise their long-term average energy intake should always accept prey items into their diet with a profitability (energy intake divided by handling time) higher than their long-term average intake rate. In a classic study, Elner & Hughes [6] used the shore crab, *Carcinus maenas,* feeding on the mussel, *Mytilus edulis,* to test the predictions of Optimal Diet Theory. They showed that when prey availability was unlimited, crabs chose mussel sizes close to the predicted optimum, but that as the optimal mussels become depleted, crabs chose progressively less valuable mussels, both above and below the optimal size. However, it can be argued that such laboratory studies of prey choice explore only the “fundamental foraging scope” [7], where foraging choices are made in the absence of most biological constraints which would be found in nature. Numerous studies have shown that these constraints can be very important in determining diet choice, for example: digestive constraints cause large mammalian herbivores to select for digestive quality over quantity [8], [9]; learning, recognition time and prey misidentification may affect energy maximisation [10]; and risk of predation often results in a trade-off between energy return and predator avoidance or vigilance, resulting in sub-optimal prey being eaten [11]–[15].

Another constraint that has been shown to affect foraging behaviour is competition. Classical optimal foraging theory predicts that competition should make foragers less choosy, as the forager cannot afford to wait for a higher value food item [16]; thus preventing some individuals from achieving their optimal diet [17]. For example, sub-optimal prey has been shown to sometimes be chosen by the three-spined stickleback, *Gasterosteus aculeatus*, when faced with interspecific competition [18]. However, in the same study, less successful individuals did not feed unselectively, as conventional diet theory would predict, but had a partial preference for smaller individuals, possibly explained by individuals learning to refrain from attacking a high quality prey for which they are likely to be outcompeted. It was argued that this strategy reduced the amount of time and energy that inferior competitors may otherwise waste [18]. Similarly, Competition Theory suggests that predators should alter their attack probabilities when faced with competitors; for example, avoiding attacking prey which is the preferred type of its opponent [19], [20], or increasing attack tendency, resulting in a broader range of prey items in the diet, as seen in juvenile the coho salmon, *Oncorhynchus kisutch*, in the presence of a simulated competitor [21].

Elner & Hughes [6] showed that when tested singly, *C. maenas* chose the predicted optimal size of prey, but intraspecific competition for food may have profound effects on their behaviour and prey selection in natural situations. Indeed, others have confirmed that interference through time lost in agonistic interactions resulted in a reduction in foraging time and overall feeding rate, demonstrating that the effects of competition were greatest under symmetric competition when crabs were sized-matched [22]. Despite this, the foraging rate of solitary individuals has not been compared with those exposed to the threat of competition, nor have the individual components of handling time been measured. Therefore, we modified the methods used by Elner & Hughes [6] to investigate the effects of competition on prey handling rates of *C. maenas*.

## Methods

Shore crabs (*Carcinus maenas*) were collected, using baited drop-nets, from the estuary of the River Plym, Plymouth, UK (50.3686, –4.1076) in October and November 2011. The crabs selected were all undamaged males with a carapace width of 6.2 to 7.6 cm (mean  =  6.82 cm, se  =  0.084). Mussels (*Mytilus edulis*) measuring 2–3 cm were collected throughout October 2011; regular collection prevented possible changes in caloric content through mass loss while held in starvation throughout the feeding trials. All mussels were collected from a single location; Queen Anne’s Battery, Plymouth, UK (50.3652, –4.1314), thereby eliminating any potential differences in shell morphology found between allopatric populations [23]. No permits or authorisation were required to collect these animals or to access the sites.

Each crab was kept in an individual aerated Perspex tank measuring 20×30×20 cm (12 L). Seawater was replaced every two days and kept at a constant ambient temperature of 15±1°C in a 12∶12 hour light-dark cycle. Newly caught crabs were fed on a diet of mussels for one week prior to the feeding trials, to ensure that all crabs had previous experience with this prey type [24]. To standardise for hunger levels, and to ensure motivation to forage, crabs were food-deprived for two days before feeding trials took place. Shore crabs can survive for three months without food [25], hence, this short period of food deprivation is sufficient to make them hungry without adversely affecting their health.

### Ethics statement

We adhered to the ASAB (2012) “*Guidelines for the treatment of animals in behavioural research and teaching*” published in Animal Behaviour 83: 301–309. No additional licensing was required for this work. No crabs died during the experiments and all were returned to the sea following the trials.

### Feeding trials

Twenty individual crabs were exposed in a random order to two trials with no competitor (NC) and two trials in the presence of competitor (C). An individual crab was placed in a tank measuring 20×30×20 cm with 12 L seawater at 15±1°C, and allowed to acclimate for at least five minutes. The feeding trial was initiated when a mussel was lowered into the tank. During competitor trials, an individual crab was then placed in the tank with another crab of similar size. We used size-matched crabs because equal-sized competitors have been shown to have the greatest impact on foraging behaviour [22]. Because we were interested in the effect of the threat of a competitor on foraging decisions, the two individuals were separated by a wire mesh, allowing chemical and visual cues to the presence of a competitor, but preventing physical contact. As in NC trials, the crabs were allowed an acclimation period and the trial was initiated when a mussel was lowered into the water on the side of the focal individual. Each feeding trial was observed from a distance and with minimal movement, so as not to disturb the crab’s feeding behaviour.

In all feeding trials, we recorded the following:

1. Breaking time (*Tb*). Defined as the time from the crab’s initial contact with the mussel, until the first bite of exposed flesh was taken; this includes recognition and manipulation, through to the crushing of the shell.
2. Eating time (*Te*). Defined as the time from the first bite of exposed flesh through until all mussel flesh was eaten. This includes re-manipulation by more shell crushing.
3. Handling time (*Th*). Defined as the sum of *Tb* and *Te*.

### Mussel energy content and Prey Value

Following Elner & Hughes [6], we calculated the energy content (*E*) of mussels using the following regression equation: *In E* (kJ)  = 3.03 *In* length (cm) – 2.34. Prey value, or intake rate, was calculated as *E/Th*.

### Statistical analyses

We tested for differences between trials and treatments in mussel size and mussel energy content using a General Linear Model to fit a two factor ANOVA. For all analyses of crab foraging behaviour we used a repeated measures General Linear Model procedure. Trial and Treatment were entered as within-subject factors and there were no between-subject factors. Before all analyses, we used one-sample Kolmogorov-Smirnov tests to confirm that the data were normally distributed. In the analyses of mussel size and energy we used Levene’s test to ensure homogeneity of variance and for the repeated-measures models we used Mauchly's test of sphericity to evaluate whether the assumption of sphericity had been violated. In all cases the assumptions of the statistical models was met. All analyses were conducted using SPSS 21.

## Results

### General feeding observations

Typically crabs showed some prey recognition time, manipulating the mussel in their chelae until orientated so that the minor chela held the mussel vertically and the major chela was positioned on the upper umbonal portion of the mussel; subsequently a crushing force was applied. The second most common method of feeding included prising the mussel shell apart to expose the flesh; this was used in conjunction with the first method if crushing was not enough to break the shell completely. These methods were similar to those described previously [26]. Prior to handling the prey, we often observed agonistic displays between the competing individuals, in the form of meral spread. Once the prey item was picked up, the individual handling this prey generally stopped such displays.

### Mussel size and energy content

Mussels used in the trials ranged in length from 2.07 – 3.00 cm (mean length: NC1  = 2.51 cm, NC2  = 2.52 cm, C1  = 2.52 cm, C2  = 2.58 cm). There was no significant difference in mussel length between trials (*F*_1,76_  = 0.483, *P* = 0.489) or treatments (*F*_1,76_ = 0.432, *P* = 0.513). Consequently, mussel energy content did not differ between trials (*F*_1,76_ = 0.549, *P* = 0.461) or treatments (*F*_1,76_ = 0.205, *P* = 0.6528), with energy content of mussels ranging from 0.87 to 2.69 J (mean energy content: NC1 = 1.625 J, NC2 = 1.736 J, C1 = 1.612 J, C2 = 1.655 J).

### Breaking time (*Tb*)

**Figure 1:**
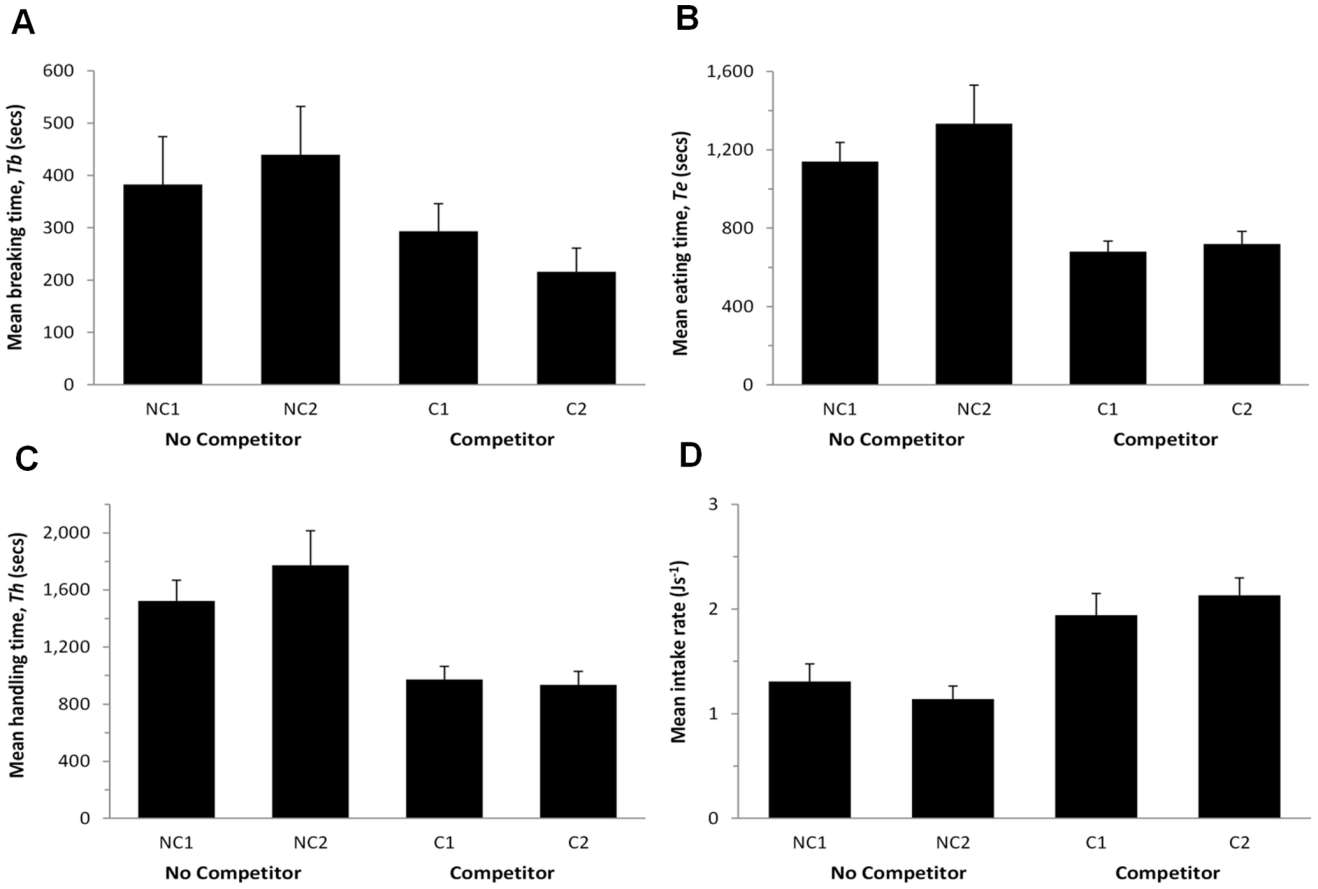
(A) Mean breaking time, *Tb* (secs), (B) mean eating time, *Te* (secs), (C) mean handling time, *Th* (secs), and (D) mean energy intake rate (Js^−1^) in each trial (1 & 2) and each treatment (NC  =  no competitor, C  =  competitor). Error bars indicate 1 standard error (*n* = 20).

In the presence of a competitor the mean *Tb* reduced by almost 40%, from 410.9 secs to 254.6 secs (*F*_1,19_  = 4.765, *P* = 0.042; Fig. 1A). There was no significant difference between Trials (*F*_1,19_ = 0.22, *P* = 0.884) and no Trial*Treatment interaction (*F*_1,19_ = 0.809, *P* = 0.380).

### Eating time (*Te*)

The effect of a competitor on *Te* was very similar to the effect on *Tb*; crabs in the competitor treatment ate far more rapidly than in the control treatment, reducing *Te* by almost 45%, from an average of 1236.7 secs to 698.8 secs (*F*_1,19_ = 20.930, *P* = 0.0002; Fig. 1B). There was no significant difference between Trials (*F*_1,19_ = 1.217, *P* = 0.284) and no significant Trial*Treatment interaction (*F*_1,19_ = 0.578, *P* = 0.456).

### Handling time (*Th*)

As *Th* is the sum of *Te* and *Tb*, the effect of a competitor on *Th* necessarily mirrors the previous results. In the competitor treatment, *Th* was approximately 42% shorter than in control treatments, with the crabs finishing their meal, on average, almost 700 seconds sooner than when there was no competitor (NC = 1647.7 secs, C = 953.4 secs; *F*_1,19_ = 20.448, *P* = 0.0002; Fig. 1C). There was no significant difference between Trials (*F*_1,19_ = 0.596, *P* = 0.450) and no significant Trial*Treatment interaction (*F*_1,19_ = 1.064, *P* = 0.315).

### Net intake rate

As a result of greatly reduced handing time, in the presence of a competitor the net intake rate of a crab increased from an average of 1.22 Js^−1^ to 2.04 Js^−1^ (*F*_1,19_ = 20.448, *P* = 0.00021; Fig. 1D). There was no significant difference between Trials (*F*_1,19_ = 0.596, *P* = 0.450) and no significant Trial*Treatment interaction (*F*_1,19_ = 1.064, *P* = 0.315).

## Discussion

This study demonstrates that competition has significant effects on feeding rates in *C. maenas*, with decreases in breaking, eating and subsequently handling times (Fig. 1 A-C). Energy intake rate, based on handling time and mussel energy content, showed significant increases when in the presence of a competitor (Fig. 1D). However, these results were counter to our *a priori* predictions. Following Elner & Hughes [6], we assumed that in the absence of a competitor, crabs would feed at an optimal rate, and that the presence of a competitor would reduce their foraging efficiency. The significant decrease in handling time in the presence of a competition, demonstrates that crabs can increase their feeding rate, raising the question of why they normally feed more slowly?

Optimal foraging theory suggests that an individual must decide on its foraging strategy based on the likely costs and benefits of the feeding attempt. These costs and benefits were originally only considered in energetic terms, but more recently it has been shown that organisms can also factor in their own nutrition state and aversive stimuli such pain [27]. Elner & Hughes [6] considered decision-making in crabs in terms of handling time and energy gain, but crabs may also be sensitive to the risk of losing a prey item to a competitor [22], [28], predation-risk [29], [30] and the risk of physical damage to their chelae [31], [32].

Although not recorded in this study, risk of claw damage when feeding is widely reported in crabs. When attempting to crush large clams, crabs sometimes break part of their dactylus, and in some instances lose their chelipeds [33]. This can impact seriously on a crab’s long-term energy intake and fitness, as chela damage and wear has been shown to have significant detrimental effects on: feeding efficiency [34], [35]; growth and moulting ability [34]; energy storage before the mating period [33]; fighting ability and attractiveness to females [36]–[39]; and ability to defend and hold onto a pre- and post-moult female [40]. Field surveys have shown that in natural populations of *C. maenas*, the proportion of male crabs with damaged or missing chelae in mating pairs was low [41], [42], indicating that intact chelae are very important in terms of mating success and ultimately fitness.

Crabs should therefore minimise activities that potentially incur chelae damage. This is often used as an explanation for sub-optimal size selection of prey by crabs [31], [32], but our result is the first indication that crabs may also vary handling time to minimise the damage to their claws. In the absence of a competitor, handling times are comparatively long, and most of the time is spent manipulating the prey item, presumably to assess its size and strength and to orient it into a suitable position to break safely. We suggest that decreased breaking times, when in the presence of a competitor, increase the risk of claw damage, and that crabs trade-off this increased risk of damage against the potential loss of the prey to a competitor.

As with breaking time, eating time was also significantly reduced in the presence of a competitor. Risk of claw damage may also contribute to reduced eating time because *Te* includes time for further breaking of the mussel shell to access the flesh. There may, however, be other costs associated with eating more rapidly. One very widely reported phenomenon is the trade-off between predator vigilance and energy intake rate [43]. *Carcinus maenas* do appear to show vigilance for conspecific competitors [28], but although anti-predator vigilance is well-documented in the fiddler crab [44], the sole study on *C. maenas* found no evidence of this [45].

It is becoming more apparent that predators often risk injury from their prey and, as a result of the significant long-term costs, predators go to great lengths to avoid injury [46]. It is argued that, because of the risk of claw damage, molluscivorous predators optimise their long-term food intake rate leading to a prey size preference that is sub-optimal within the energy maximisation framework [28]. We suggest that this logic may be extended to apply to handling time, and that crabs typically break shells at a rate below their maximum in order to minimise claw damage. In the presence of a competitor, crabs appear to become more risk-prone and handle their food more rapidly, minimising the risk of kleptoparasitism.
